# The potential for current sodium and potassium production to support a global switch to the use of potassium-enriched salt: a desktop research study

**DOI:** 10.1017/S1368980024000922

**Published:** 2024-04-22

**Authors:** James David Bullen, Katrina Rashelle Kissock, Xuejun Yin, Penjani Mkambula, Kathy Trieu, Bradley Hastings, Bruce Neal, Ellie Paige

**Affiliations:** 1 The George Institute for Global Health, Sydney, NSW, Australia; 2 The University of New South Wales, Sydney, NSW, Australia; 3 The National Centre for Epidemiology and Population Health, The Australian National University, Canberra, ACT, Australia; 4 The Global Alliance for Improved Nutrition, Geneva, Switzerland

**Keywords:** Sodium chloride, Dietary salt, Potassium chloride, Diet, Food and nutrition, Salt substitute, Potassium-enriched salt, Salt supply, Salt industry

## Abstract

**Objective:**

Switching regular salt (sodium chloride) to salt enriched with potassium chloride (25 % potassium chloride, 75 % sodium chloride) has been shown to reduce blood pressure and the risk of cardiovascular diseases. We sought to define the potential for the current production of sodium chloride and potassium chloride to support a global switch to the use of potassium-enriched salt.

**Design:**

We summarised data from geological surveys, government reports and trade organisations describing the global production and supply of sodium chloride and potash (the primary source of potassium chloride) and compared this to potential requirements for potassium-enriched salt.

**Setting:**

Global.

**Participants:**

Not applicable.

**Results:**

Approximately 280 million tonnes of sodium chloride were produced in 2020 with China and the USA the main producers. Global production of potash from which potassium chloride is extracted was about forty-four million tonnes with Canada, Belarus, Russia and China providing 77 % of the world’s supply. There were forty-eight countries in which potassium-enriched salt is currently marketed with seventy-nine different brands identified. Allowing for loss of salt between manufacture and consumption, a full global switch from regular salt to potassium-enriched salt would require about 9·7 million tonnes of sodium chloride to be replaced with 9·7 million tonnes of potassium chloride annually.

**Conclusions:**

Significant upscaling of the production of potassium chloride and the capacity of companies able to manufacture potassium-enriched salt, as well as a robust business case for the switch to potassium chloride, would be required.

Cardiovascular diseases (CVD) are the leading cause of death worldwide^(1)^. Excessive consumption of sodium, which primarily comes from dietary salt (sodium chloride), is linked to high blood pressure and an increased risk of CVD^(2,3)^. The same is true for insufficient consumption of dietary potassium^(4)^. There has been a significant public health effort over past decades to reduce sodium consumption by decreasing salt intake and to increase potassium consumption by encouraging the intake of fresh fruits and vegetables. Both have proved difficult to achieve due to the many barriers to sustained and scalable change at the individual to the macro-environmental level^(5)^. This is despite strong advocacy from the WHO and a high likelihood of cost-effective health gains^(6)^.

Potassium-enriched salt, in which a portion of the sodium chloride in regular salt is replaced with potassium chloride, has long-established efficacy in reducing dietary sodium intake, increasing dietary potassium intake and lowering blood pressure^(7)^. Reduced sodium consumption and increased potassium consumption both have separate blood pressure-lowering effects and may be synergistic in some settings^(8)^. A recent large-scale randomised trial of 20 995 older participants done in rural China showed a 14 % reduction in stroke, 13 % reduction in major CVD and 12 % reduction in all-cause mortality among those using potassium-enriched salt compared with regular salt^(9)^. A recent review of all studies of potassium-enriched salt indicated a high likelihood that widespread use of potassium-enriched salt would be associated with important health benefits across the life course for diverse communities worldwide^(7)^.

A key advantage of potassium-enriched salt over other efforts to change dietary consumption of sodium and potassium is that it is easy to start and continue using. Potassium-enriched salt has similar organoleptic characteristics (i.e. taste, colour, odour and feel) to regular salt^(10)^. In addition, potassium-enriched salts can be cost-saving and cost-effective^(11)^. There is also a clear precedent for intervening to change the world’s salt supply to address population health issues, with highly effective salt iodisation programmes implemented worldwide to address iodine deficiency disorders^(12)^.

The primary aim of this paper is to summarise data describing the production and supply of the core constituents of potassium-enriched salt (sodium chloride and potassium chloride) with a view to assessing the medium-term (10–25 year) feasibility of switching the world’s salt supply from regular iodised salt to potassium-enriched iodised salt.

## Methods

### Data sources

We sought summary data sources describing global salt and potash production and use, including quantities produced, production methods employed and end uses (including consumption and use in the food industry). Data were sought from the British Geological Survey, the United States Geological Survey, multiple other government reports, research papers and country-specific organisations^(13–16)^. Data describing the major companies involved in production in each country were sourced from these reports with additional information on companies involved in the salt industry obtained using industry classification system codes – for example, the North American Industry Classification System and the Standard Industrial Classification codes assign unique identifiers to individual industries which enabled us to search business databases Company360 and Business Source Ultimate^(17–20)^. To locate these data, we searched high-quality medical and other databases including PubMed, Web of Science and Scopus using keywords relating to the topic area (potassium-enriched salt, salt substitute, sodium chloride, sodium reduction, low-sodium salt, sodium production, potassium production and related synonyms). We also performed the same keyword searches using the Google search engine and Google Scholar.

Data on the production of sodium chloride and potash by country and year for the period 1990–2020 were obtained from the British Geological Survey. Data on the import and export of sodium chloride and potash by country and year for the period 1990–2020 were obtained from the UN Comtrade database, which collects comprehensive trade statistics by commodity and country^(21)^. Commodity code 2501, the UN Comtrade code for all sodium chloride, including table salt and denatured salt, was used. There was no specific code for food-grade sodium chloride identified. Commodity code 310420, the code for potassium chloride for use as a fertiliser, was used for potash since there is no specific code for potassium chloride or food-grade potassium chloride. Data on the production of potassium-enriched salts by country were obtained from our previously published work, in which potassium-enriched salts were identified through a systematic review of the literature, search engine and shopping website searches and key informant interviews, to develop a database recording key product information, including product name, brand name, country, composition, labelling information and price^(22)^.

### Analysis

We identified and ranked the ten countries with the highest reported mean annual production for sodium chloride and potash. Data describing the major companies producing sodium chloride and potash were summarised in table and narrative forms. The availability of potassium-enriched salt products in each country was extracted and summarised using information held in the database described above^(22)^. To estimate current global human requirements for sodium chloride, we assumed a mean global intake of 10 g/d per person^(23)^ multiplied by the average number of days in a year (365·25) by the global population (eight billion people)^(24)^ and adjusted to allow for 33 % loss and wastage during salt manufacture, food production and consumption^(25)^. Projected requirements for sodium chloride and potassium chloride if the global salt supply were switched to potassium-enriched salt were estimated by reducing sodium chloride requirements by one-quarter (the same proportions as used in the large-scale randomised salt substitute and stroke study)^(9)^ and using that number as the estimate of the required quantity of potassium chloride.

A global switch using the proportions 25 % potassium chloride and 75 % sodium chloride was selected for a number of reasons. The weight of scientific evidence for the health benefits of a switch to a potassium-enriched salt comes from studies where a 25:75 blend has been used. Typically, potassium-enriched salt blends containing 25–30 % KCl have been found to be highly acceptable to consumers, and they are less able to tell the difference between the product and a regular salt, key to the feasibility of a global switch^(10)^. There is also a lower theoretical risk of hyperkalemia when using blends with a lower proportion of potassium chloride (as the total amount of potassium an individual consumes is lower). However, there are other possible blends of potassium-enriched salt that may be desirable in specific contexts, with many such products available on global markets today^(22)^.

Data analysis and visualisation were performed using R version 4·2·0 and RStudio 2022·02·3 + 492 ‘Prairie Trillium’ release for Windows^(26,27)^.

## Results

### Sodium chloride production and trade between countries

**Fig. 1:**
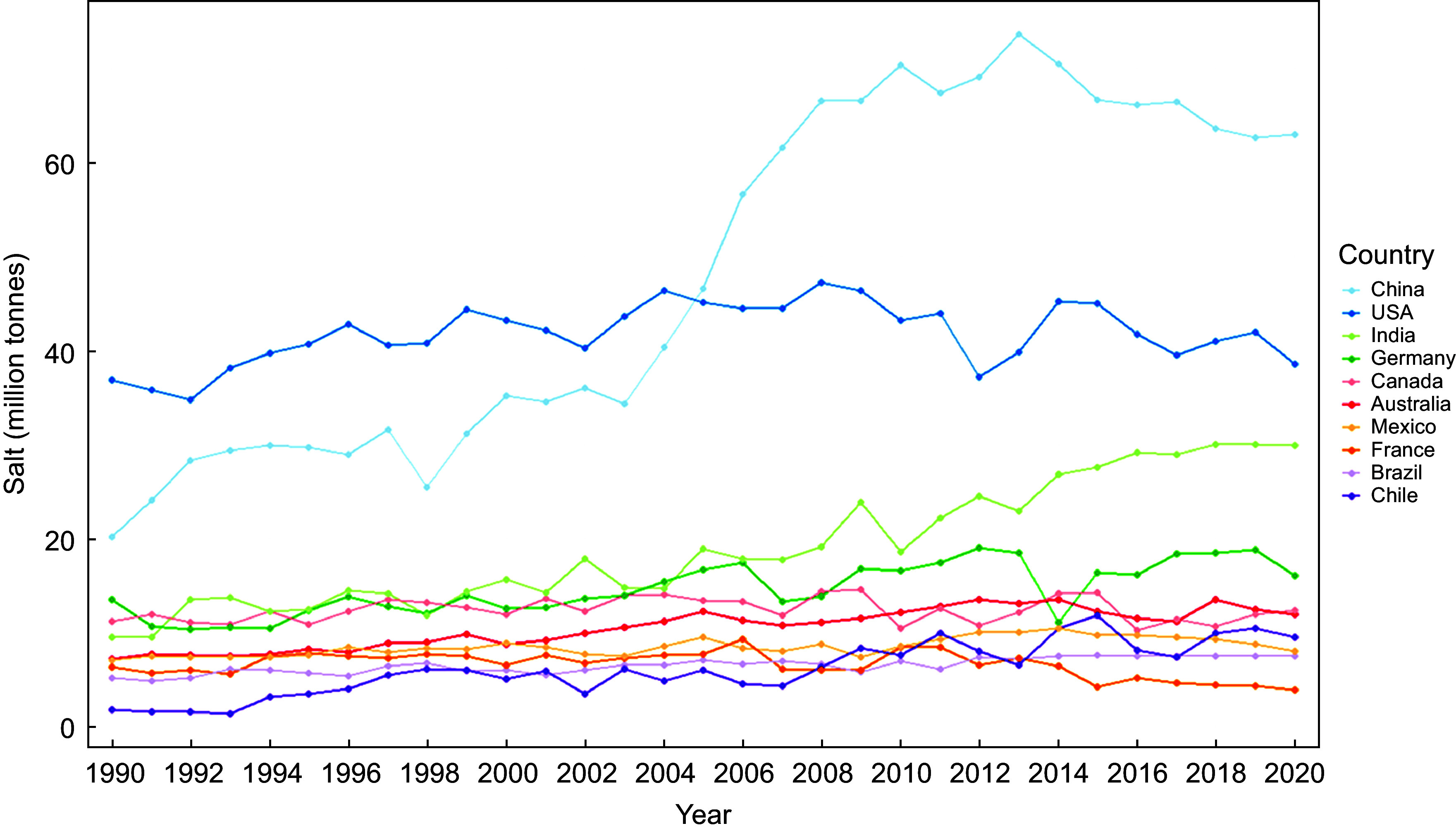
Sodium chloride produced by ten countries leading manufacturing (1990–2020). Source: World Mineral Statistics (British Geological Survey)

**Fig. 2:**
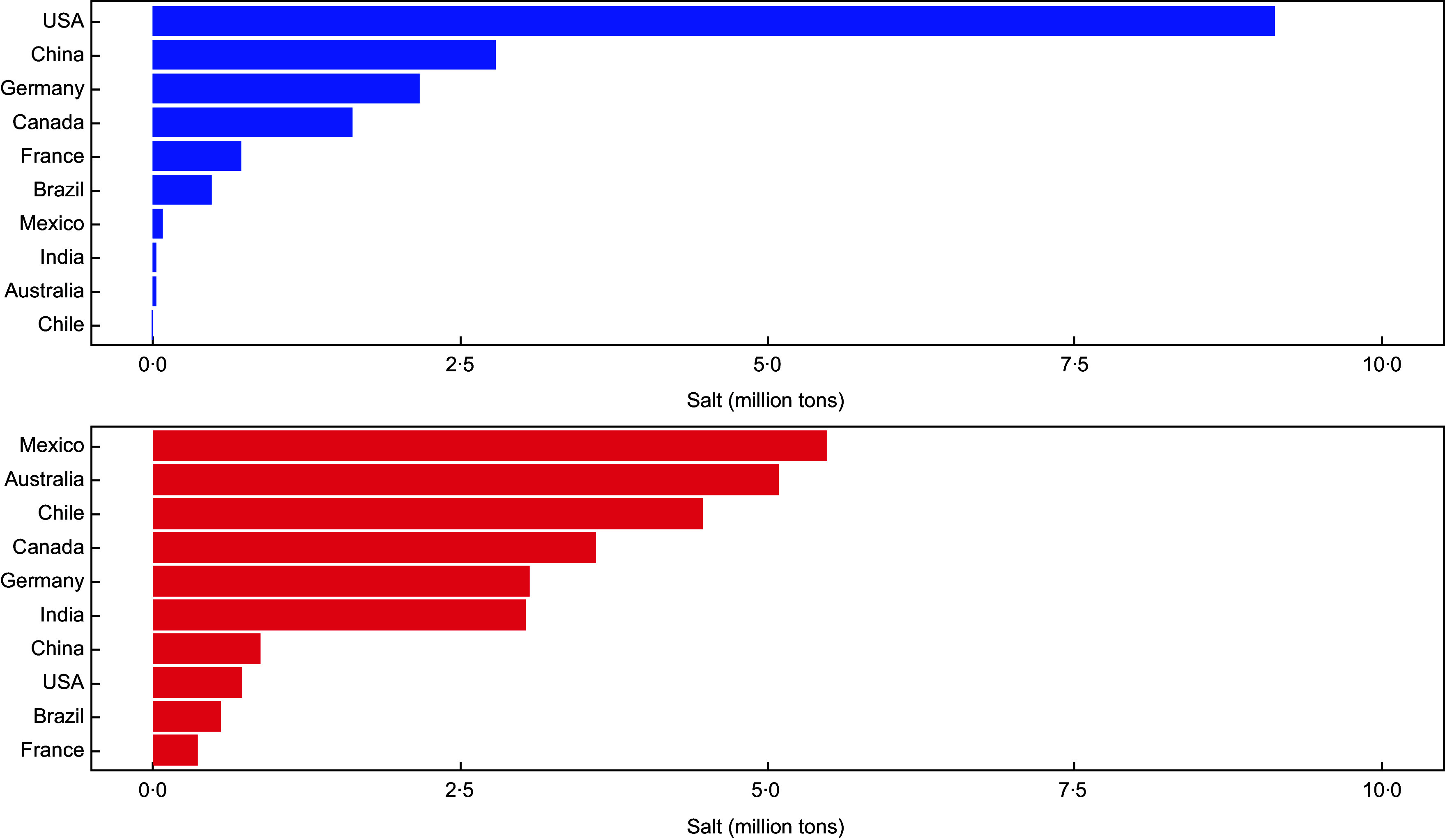
Mean annual import (top) and export (bottom) of sodium chloride for ten leading countries (1990–2020). Source: UN Comtrade

Global production of sodium chloride in 2020 was estimated to be 280 million tonnes which increased by 86 % from an estimated global production of 145 million tonnes in 1990 (Fig. 1). The three leading manufacturers in 2020 were China (63 million tonnes, 22·8 %), the USA (38·6 million tonnes, 14 %) and India (29·9 million tonnes, 10·8 %) with all other countries each producing less than 20 million tonnes annually. Growth in annual global production has been driven predominantly by China and India. For the period 1990–2020, the USA (nine million tonnes), China (three million tonnes) and Germany (two million tonnes) were on average the largest importers of salt worldwide, while Mexico (five million tonnes), Australia (five million tonnes) and Chile (five million tonnes) were the largest exporters (Fig. 2).

### Sodium chloride production methods

Sodium chloride is produced using four main methods. Rock salt can be mined as a solid product from underground deposits, extracted as a salt brine from underground deposits by injecting a water-based solvent, by solar evaporation of salt water and by artificially heated evaporation of saltwater in vacuum pans^(16)^. Production methods vary by geography and the form of local resources, with the majority of salt production in Canada, China and the USA deriving from mined rock salt and almost all salt produced in Australia and India manufactured through solar evaporation of seawater^(14,16,28,29)^. Since sodium chloride can be obtained from seawater global reserves are effectively unlimited. Vacuum pan and solar evaporation methods are more expensive compared with mining methods with 2021 production costs of US$220 per tonne for vacuum pan, US$120 per tonne for solar evaporation, US$56 per tonne for conventional rock salt mining and US$9 per tonne for brine salt mining^(30)^. A significant proportion of the world’s salt supply originates from a small number of major salt manufacturers. A smaller, but still significant, proportion of supply comes from numerous small-scale independent producers. This is especially the case in low- and middle-income countries^(12)^.

Food-grade sodium chloride usually comes from the solar evaporation or vacuum pan production methods because rock salt typically contains impurities that require significant processing to remove. The solar evaporation and vacuum pan production methods enable contaminating minerals like magnesium and calcium to be removed by passing the brine through a series of evaporation ponds from which concentrated, crystallised sodium chloride with a purity above the 99 % required for food-grade sodium chloride can be obtained^(31,32)^.

### Main uses for sodium chloride

Robust quantitative data describing the end use of salt were absent for most jurisdictions, but salt use varies considerably across geographies. De-icing in cold climate countries, industrial chemical processes in major manufacturing economies and agriculture are leading uses^(13,16)^. Salt for human consumption accounts for only a small proportion of salt use in countries that are large producers or importers. Food-grade sodium chloride is used in food processing as a flavour enhancer, preservative, binder and for texture^(33)^.

### Potash production and trade between countries

**Fig. 3:**
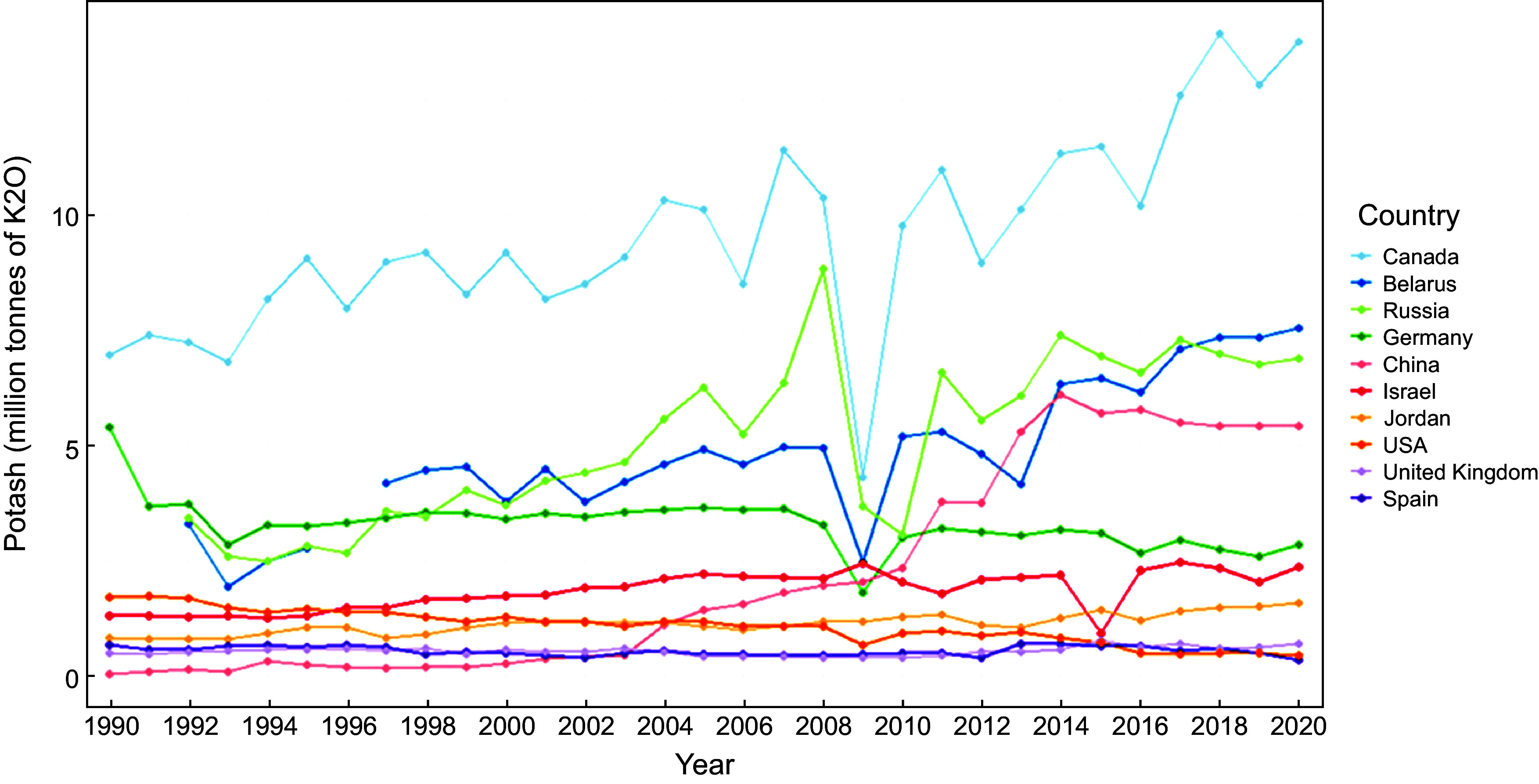
Potash produced by ten countries leading manufacturing (1990–2020). Source: World Mineral Statistics (British Geological Survey)

Potash is the umbrella term used to describe the materials from which the global supply of potassium is obtained. Most of the world’s potash is in underground deposits, which formed when ancient inland oceans evaporated and the potassium salts they contained were crystallised^(34)^. Potash holds potassium in multiple chemical forms including potassium chloride (KCl), potassium sulfate (K_2_SO_4_) and potassium nitrate (KNO_3_). Quantification of the potassium content of potash is usually expressed in units of equivalent potassium oxide (K_2_O) to provide a standardised basis for comparison between sources. This is because different forms of potash have different amounts of potassium, and expressing in K_2_O provides a common denominator across industries^(35)^. Global potash production in 2020 was estimated to be forty-four million tonnes which has increased by about a half from 1990 (Fig. 3), with global reserves of potash estimated at approximately 3·5 billion tonnes^(30)^. These are reserves that are considered technologically and economically feasible to recover, or what is termed in the mining/resources industry a ‘recoverable resource’ or ‘recoverable ore’^(30)^. A recent paper estimates global potash production may peak in 2057 at 55·3 million tonnes^(36)^.

**Fig. 4:**
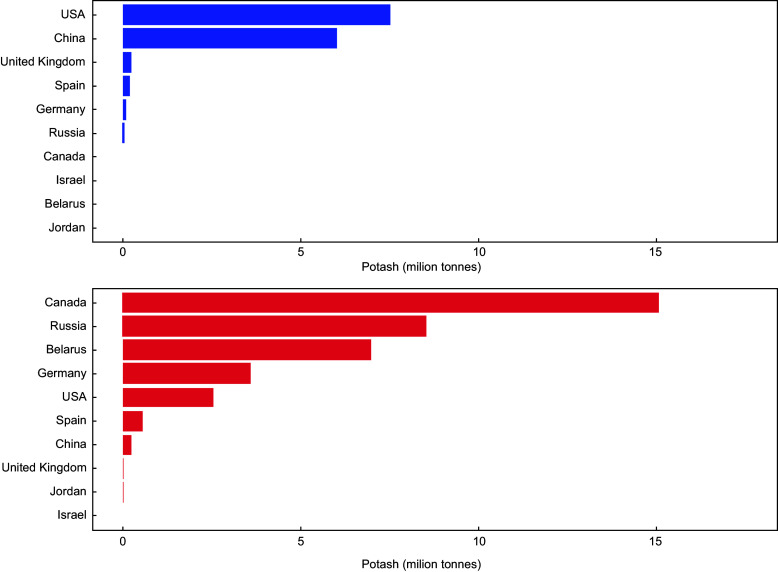
Mean annual import (top) and export (bottom) of potash for ten leading countries (1990–2020). Source: UN Comtrade

Four countries produced more than three-quarters of the world’s potash – Canada (13·8 million tonnes, 31 %), Belarus (7·6 million tonnes, 17 %), Russia (6·9 million tonnes, 16 %) and China (5·5 million tonnes, 12 %) in 2020 (Fig. 3). All other countries produce less than four million tonnes a year. Canada, Russia and Belarus are the main exporters, and the USA and China are the main importers (Fig. 4). The Russian war in Ukraine has constrained potash supply and led to fluctuating global prices^(37)^.

### Potash production methods

Most potash production is from traditional underground mining of ore deposits several hundred metres below the Earth’s surface^(38,39)^. Small quantities are also obtained using solution mining techniques or from the evaporation of surface-level potassium-rich water bodies^(39)^. Food-grade potassium is typically manufactured using an evaporation process that, as for food-grade sodium chloride, allows for the removal of impurities and a high-purity crystalline product^(40)^. It is unknown what proportion of the world’s reserves are available via the evaporation or solution mining means of production.

### Main uses for potash

Approximately 90 % of the world’s potash production is used for fertiliser products with the remainder used mostly in the manufacture of pharmaceuticals, detergents and water conditioners^(16,35)^. Small quantities are used in food applications such as potassium-enriched salts and maize-soy blends used in malnutrition programmes^(41)^.

### Potassium-enriched salt and potential for scaling

Potassium-enriched salt products are a fraction of the total available salt products in the global marketplace (Table 1) and are typically marketed as a healthy alternative to regular table salt^(22)^. Previous work has identified higher production costs compared with regular salt and limited market demand as key barriers to the scaling of potassium-enriched salt use while noting health education, mass media campaigns and price control as potential levers for increasing uptake^(42)^.

**Table 1:** Ten countries manufacturing most sodium chloride and availability of potassium-enriched salts

	Estimated annual sodium chloride production (million tonnes)	Major salt-producing companies	Potassium-enriched salt brands	Number of potassium-enriched salt products on market
China	48·3	China National Salt Industry Corporation (China Salt)	Nu Salt, China Salt, Yue Salt, Hai Yuan Wei, Hai Jing, Qinghai Salt, Huai Salt, Ruifeng Salt, Orange Tree, Jiu Da, Luhua, Qi Jia, Henan Salt, Xuetian, Yi Yantang, Zhonghuyan	21
USA	41·8	Cargill, Morton International	Nattierra, Salt for Life, Aromasong, Dr Murray’s, Morton	5
India	19·1	Tata Salt	Dr Salt, Nutroactive, Tata Salt	4
Germany	14·7	K + S Minerals and Agriculture GmbH, Südwestdeutsche Salzwerke AG	Dr Jacob’s	1
Canada	12·4	Canadian Salt Company Limited, Sifto Canada Inc.	Windsor	1
Australia	10·6	Cheetham Salt, Shark Bay Salt, Dampier Salt	Diet Rite, Heart Salt	2
Mexico	8·6	Exportadora De Sal S.A, Sales Del Itsmo, Pennwalt	La Fina	1
France	6·6	Groupe Salins, Quadrimex Sels	La Baleine, Castello	2
Brazil	6·6	Salinor, Cimsal	Sosal	1
Chile	6·1	Seromin Chile SpA	Lobos	1

A global switch from the use of regular salt to potassium-enriched salt comprising 25 % potassium chloride and 75 % sodium chloride would require about 9·7 million tonnes of potassium chloride each year. This figure is based on the estimated current annual salt consumption of 29·2 million tonnes (average 10 g/d for each person in the world) and allows for 33 % wastage between manufacture, food preparation and consumption (estimated total annual sodium chloride sales of 38·9 million tonnes)^(25)^.

While precise figures are not available for total food loss (the decrease in quantity or quality of food along the supply chain, from the point of production to the point of retail sale) and food waste (disposal of food that can be used, usually by retailers and consumers) for salt, global estimates suggest one-third of food produced for human consumption is lost and wasted and we adopt the same figure here^(25)^. It is, however, important to note that per capita food loss and waste vary by region and country income, with per capita food wasted by consumers in North America and Europe at 95–115 kg/year while consumers in sub-Saharan Africa and South/Southeast Asia only wasted 6–11 kg/year^(25)^.

This estimated 33 % of food produced for human consumption being lost or wasted was determined in two studies conducted by the Swedish Institute for Food and Biotechnology. One study focused on medium- and high-income countries and the other on low-income countries. It used total food volumes and food balance sheets from the FAO, as well as data in the literature to calculate food lost and wasted. These studies did not specifically consider salt as a food item in their calculations. However, given salt sees significant use in food processing and as a food ingredient, it is likely that general food loss and waste are responsible for a significant proportion of salt loss and waste in the food supply.

9·7 million tonnes of potassium chloride constitutes about one-fifth (9·7/44 million tonnes) of current annual potash production. A global switch to potassium-enriched salt would result in a corresponding reduced demand for sodium chloride of 9·7 million tonnes, which equates to about 3·5 % (9·7/280 million tonnes) of current annual production.

Incomplete switching of the salt supply would require proportionately lesser differences to the manufacture of sodium chloride and potassium chloride. For example, the replacement of 10 % of the salt supply would require about one million tonnes of potassium chloride, would have negligible impact on sales of sodium chloride and is perhaps a more likely scenario for a 5- or 10-year time frame.

## Discussion

The potential for global health gains from switching the world’s salt supply to potassium-enriched salt is large, but this paper identifies significant supply chain challenges that will need to be resolved if these benefits were to be realised. Specifically, while the reduced requirement for sodium chloride following a global switch to potassium-enriched salt constitutes only a small proportion of the total global production of sodium chloride, there will be a large increase in the quantity of potassium chloride required. Increasing potassium chloride production and, in particular, increasing the supply of food-grade potassium chloride will require a major expansion of industry capacity. This will require significant investment, a protracted time frame and a strong business case for successful implementation.

Potassium chloride is more expensive than sodium chloride, which means changing the economics of the potassium-enriched salt end product will be key. That will likely involve improving economies of scale for potassium chloride production to lower production costs and identifying other mechanisms that minimise the cost to end users. While the supply chain changes required to achieve full global switching of the salt supply are significant, in practice, they are likely to take many years and be done piecemeal across jurisdictions and industries which will make them more feasible. An additional challenge is that smaller, fragmented and artisanal industries are the majority of salt production in some parts of the developing world, and this will present an additional barrier to the replacement of sodium with potassium chloride^(12)^.

Global reserves of potash are large, so increased production of potassium chloride would appear feasible. Presently, the extraction of potash is concentrated in a few countries with large-scale mining facilities, and it is likely that current production is focused on the most easily accessible and lowest-cost reserves. The capacity to increase production from existing facilities is unknown, and the extent to which economies of scale might offset the investments required to increase potash production are unclear. A specific current challenge to the global production of potash is the Russian war in Ukraine and associated sanctions from Western nations, which have led to reduced exports from Russia. This has in turn resulted in a global shortage of fertiliser products and greatly increased prices for potash in 2022 compared with 2021^(37)^. The outlook for potash demand remains strong because growth in the world’s population is driving a requirement for fertilisers that can increase agricultural output, particularly in the populous regions of Southeast Asia, Africa and Latin America^(38)^. While it is hoped that current supply chain constraints will be resolved in the short term, volatility in prices will likely be a challenge for investors.

The broader commercial factors that will affect a switch from the manufacture and supply of regular salt to potassium-enriched salt are multiple. The proportional decrease in demand for sodium chloride will be small as a proportion of total production but may have a disproportionate impact on the industry because food-grade sodium chloride is a higher-value product than the sodium chloride used for purposes such as de-icing. Switching to a product that requires less food-grade sodium chloride may therefore be unwelcome and resisted by incumbent providers. At the same time, there may be an incentive for potash producers and refiners to increase production because food-grade potassium chloride could become a new high-value output from their operations. Increasing potash production and building the capacity to refine food-grade potassium chloride will, however, require significant capital investment. In parallel, current manufacturers of food-grade salt may view potassium-enriched salt as a market opportunity whereby current sales of regular salt can be replaced with a higher-value product upon which an increased profit margin can be achieved. A switch to potassium-enriched salt would however require retooling of manufacturing facilities with requirements for capital investment.

The current market for potassium-enriched salt products is small, though a few countries like China, India and the USA have multiple product lines available. Potassium-enriched salts are variously produced by specialist companies or the existing manufacturers of regular salt. A key question for the scale-up of potassium-enriched salt use will be whether the opportunity lies in improving the position and capacity of existing potassium-enriched salt production companies, which presently serve only a tiny fraction of the global ‘salt’ market, or in persuading existing large-scale manufacturers of salt and high-sodium products such as seasonings or bouillon cubes to add potassium-enriched salt to their product lines^(43)^.

As for other forms of extraction mining, there are concerns over the environmental impact of potash mining, which can include damage to topsoil, vegetation, surface and groundwater. There are also potential serious adverse environmental effects of shipping large volumes of potash around the world given the current concentration of production in a few countries^(39)^. These environmental impacts may become a limiting factor as the trend towards rigorous and systematic reporting for manufacturers and retailers continues^(44)^. Further, the global distribution of potash reserves and the commodity’s price may make access for some countries, especially those in Africa, a challenge^(38)^. Other economic factors including currency volatility and depreciation can lead to fluctuations in the price of imports and local production costs^(45)^.

A significant limitation to this report is that much better data are needed to define the manufacture, import and export of sodium chloride and potassium chloride. Improvements to data would include being able to determine the end use of salt production by country (i.e. whether it is used for food, industrial, chemical or other purposes) and means of production (i.e. whether it is produced via rock salt mining, salt brine extraction, solar evaporation or vacuum pan methods). Some of this information is currently available, but it is patchy and country-dependent. These improvements would enable a clearer picture of global production and supply as relevant to food systems. While both food-grade sodium chloride and food-grade potassium chloride constitute only a small proportion of total production, the factors influencing their markets are likely to be different from the factors influencing the market for overall production. The USA is one of the few countries to provide detailed accounts of its use of sodium chloride through annual reports, but even these reports provide limited insight into food-grade product^(16)^. There are currently no specific industry commodity codes for food-grade sodium chloride and potassium chloride, which makes a more granular analysis of the production and use of these commodities impossible at present and means the estimates provided in this study may not be precise.

A related limitation is that the estimated production requirements of potassium chloride and sodium chloride for a switch of the world’s salt supply from the use of regular table salt to a 25:75 potassium-enriched salt are likely to be imprecise. This is particularly the case given estimates for food loss and food wastage used in this paper are themselves broad in nature and do not specifically consider salt loss and wastage.

### Conclusion

Given the many challenges in making even a partial switch of the salt supply to potassium-enriched salt, work to define the different possible pathways to market will be key. It will be necessary to construct robust business cases and the scaffolding that companies can use to deliver potassium-enriched salt alternatives for consumers in diverse settings. Prior studies using data from China and Vietnam have demonstrated that replacing regular salt with potassium-enriched salt is a cost-effective intervention, another important consideration for policymakers when evaluating the implementation of potassium-enriched salt scale-up^(11,46)^. There is an additional need to define the associated policy and advocacy efforts that will communicate the public health case for incorporating potassium chloride into product lines for large manufacturers of salt, seasonings and bouillon. The latter is particularly important in many lower-income settings where these products contribute a significant component of daily salt intake^(47)^. It will be useful to consider pathways to market for other products and commodities that have shifted in their purpose and objectives over time and that have associated health benefits, such as fortified salt and spreads with added plant sterols that sit alongside traditional food products such as table salt and butter^(48,49)^. Parallel efforts to explore the potential for subsidy or prescription of potassium-enriched salt through health systems, particularly for those at risk of CVD may be an effective way of enhancing consumer acceptance and improving access. Case studies of pathways to implementation for a series of countries that define what would be required to scale up, taking into account production changes needed at salt processors, market structures, the prevalence of diseases and health and economic outcomes, would all be valuable. Specific country case study opportunities include China, which is planning to scale up the substitution of potassium chloride in salt products, and Singapore, where the country’s health promotion board has been advocating for the adoption of potassium-enriched salt products and other reduced-sodium salts, as part of a broader strategy of sodium reduction^(50)^.

There are ample global reserves of potassium chloride to support a switch to the widespread use of potassium-enriched salt, but there will be major changes to production and manufacturing capacity required. This will include a requirement for expanded mining of potash substrate, greatly increased capacity for refining food-grade potassium chloride and a major switch to the manufacture, marketing and sale of potassium-enriched salt products. Further research into pathways to market for potassium-enriched salts could provide useful evidence of their feasibility.
